# Facilitators, barriers and service availability for delivering integrated care for the triple elimination of HIV, syphilis and hepatitis B vertical transmission in Uganda: a multi-site explanatory mixed methods study

**DOI:** 10.1186/s12913-025-12797-4

**Published:** 2025-05-01

**Authors:** Andrew Kazibwe, Emmanuel Olal, Andrew Mijumbi Ojok, John Vianney Kigongo, Henry Kafumbe, Maria Prima Niwampeire, Charity Gloria Toskin, Doreen Ondo, Linda Kisaakye Nabitaka, Patience Mwine, David Kagimu, Anna Lawino, Michael Bernard Etukoit

**Affiliations:** 1 The AIDS Support Organisation (TASO), Mulago Hospital Complex, P. O. Box 10443, Kampala, Uganda; 2 Clinton Health Access Initiative (CHAI), Plot 8, Moyo Close, Kampala, Uganda; 3 Makerere University College of Health Sciences, P. O. Box 7072, Kampala, Uganda; 4 Elizabeth Glaser Pediatric AIDS Foundation, P. O. Box 21127, Kampala, Uganda; 5 AIDS Control Division, Ministry of Health, Lourdel Road, P. O. Box 7272, Kampala, Uganda

**Keywords:** Triple elimination, Vertical transmission, Service integration, HIV, Syphilis, Hepatitis B, Health systems

## Abstract

**Background:**

Elimination of vertical transmission of HIV, syphilis and hepatitis B is part of the global aspiration to end the three infections as public health threats by 2030. Whereas global and national policy guidelines recommend integration of screening, prevention and treatment for the three infections in maternal and child health (MCH) service delivery points, progress has been slow. We aimed to explore the health system factors that facilitate and hinder optimal integration of triple elimination services within the MCH platforms.

**Methods:**

This was a cross-sectional, explanatory mixed methods multi-site study implemented in two regions of Uganda, conducted in July – August 2024. Firstly, we used an observation checklist to assess for the availability of services and commodities required for provision of triple elimination care at 20 health facilities (two regional referral hospitals, two general hospitals, two specialized outpatient TASO clinics, five HCIVs, eight HCIIIs and one HCII), and computed a percentage service and commodity availability score for each site, and average for the sites. We then used findings from this assessment to guide open-ended probing during key informant interviews and focus group discussions among ten key informants and 43 focus group discussion participants. Interviews and discussions were recorded, transcribed verbatim, and then analysed manually. We categorized responses as either facilitators or barriers and extracted quotes, by theme, based on the World Health Organization’s health systems building blocks framework.

**Results:**

The average percentage score of service and commodity availability was 61.8% (range: 46.4–78.6%) in Acholi region and 66.1% (range: 53.6–78.6%) in Teso region. We found that presence of trained focal persons, district accountability fora, routine data collection and utilization, and availability of motivated community health workers facilitated triple elimination service integration. Key barriers included limited district health team engagement, frequent stock-outs of diagnostic and treatment commodities, health personnel shortages and high reporting burden.

**Conclusions:**

Health facility service readiness and availability percentage scores differed across facilities and between the two regions. Several health system factors facilitate integrated service provision for elimination of HIV, syphilis and hepatitis B vertical transmission. This integration is, however, constrained by a number of health system barriers. Further implementation research could contribute to addressing the various health system constraints and adoption of strategies for service integration tailored to site contexts.

**Supplementary Information:**

The online version contains supplementary material available at 10.1186/s12913-025-12797-4.

## Background

Human immunodeficiency virus (HIV), chronic hepatitis B virus (HBV) infection, and syphilis are prioritised for elimination by 2030 [1-3]. Yet, there were 1.3 million new HIV infections in 2023, and this is more than triple the 2025 target of less than 370,000 per year, globally. In the same year, there were over 630,000 deaths attributable to HIV, more than double the global elimination target of less than 250,000 per year. In 2022, there were over 1.4 million individuals newly infected with HBV, and at least 250 million people were estimated to be living with Chronic HBV. Finally, the World Health Organisation (WHO) estimates that there were over eight million individuals with syphilis in 2022, higher than the 7.1 million people in 2020. Vertical transmission, from mothers to their children, during pregnancy, child-birth and breastfeeding, contributes to the high number of new infections.

More than 10% of new HIV infections and 90% of HBV infections are due to vertical transmission [3, 4]. Likewise, the World Health Organization (WHO) in 2022 estimated that over 700,000 cases of congenital syphilis occurred, resulting in nearly 150,000 perinatal deaths (early fetal deaths and still-births) [5, 6]. The shared transmission pathways of HIV, hepatitis B infection and syphilis provide an opportunity to avert this burden of disease and human suffering through integrated services, targeting pregnant and lactating mothers, and their infants in maternal child health clinics. While affordable and high-quality tools for testing, treatment, and prevention commodities are globally available, their access among pregnant women and infants remains uneven, leading to the persistently high incidence of vertical transmission in low- and middle-income countries (LMICs), particularly in Sub-Saharan Africa (SSA) [1, 4]. Thence, there is a growing need to explore delivery of triple elimination services within the MCH platforms.

The WHO and the global community have committed to targets to eliminate vertical transmission of HIV, syphilis and HBV, which include zero new HIV infections among children and adolescents (and attainment of 95-95-95 targets), 95% reduction in incidence of chronic HBV infections and less than 50 cases of congenital syphilis per 100,000 live births in 80% of countries [2]. While progress has been made, this is still too slow to meet the targets: nearly 120,000 children aged 0–14 years acquired HIV in 2023, far from the target of less than 20,000 per year, hepatitis B surface antigen prevalence among children younger than 5 years old was 0.94% in 2020 distant from the target of less than 0.5%, while congenital syphilis prevalence globally was 473 cases per 100,000 live births in 2023 [7-9]. Service integration is therefore expected to accelerate global progress towards attainment of validation targets, individual countries need to commit to the triple elimination agenda [3].

Service integration under the triple elimination approach involves co-locating and/or bi-directional referral for HIV, hepatitis B and syphilis screening using rapid diagnostic kits, treatment and infant vaccination with hepatitis B birth dose vaccines in maternal and child health care points for optimum service delivery [4, 5]. A survey found that while national policies supporting dual HIV/syphilis rapid diagnostic tests (RDTs) for pregnant women exist in all 16 countries in sub-Saharan Africa surveyed, the availability of these dual RDTs was limited [10]. Testing for HBV using the hepatitis B surface antigen (HBsAg) test was even less accessible, highlighting significant gaps in service delivery [10]. In Uganda, while there has been significant expansion in syphilis testing and treatment services for pregnant women since 2017, driven by the introduction of dual rapid diagnostic test kits for HIV and syphilis, hepatitis B testing integration has been slower [11]. Despite development of a national package of care and several guidelines, [12] comprehensive implementation of the triple elimination is still slow in some regions. For example, [13] Uganda Ministry of Health’s elimination of mother-to-child transmission (eMTCT) program performance review from 1st October 2020-30th September 2023 showed low coverage of Hepatitis B testing among pregnant and breastfeeding adolescents and women for Teso and Acholi subregions at 32% and 35% respectively. The performance of the two regions was lower than the national average of 50%. The performance was equally poor for hepatitis B treatment uptake at 24% and 43% in the Acholi and Teso sub-regions respectively. Moreover, the two regions have higher-than-national-average prevalence of chronic hepatitis B infection at 4.6% (Acholi) and 4.3% (Teso) compared to 4.0% (national) [14]. The HIV prevalence in Acholi region (7.6%) was also higher than the national average (5.1%). There was, therefore, a need to examine the health system facilitators and barriers, to advancing the triple elimination agenda, more-so in these regions.

We report formative findings of a multi-site study that applied the WHO health systems strengthening framework to inquire into health system facilitators and barriers to implementing triple elimination services in antenatal care clinics in Uganda. The WHO health systems strengthening framework for action defines six discrete blocks to provide a shared understanding of health systems and guide health system strengthening efforts [12]. The study utilized this qualitative inquiry to understand findings from a service and commodity availability assessment for delivery of triple elimination services at the selected health facilities.

## Methods

### Study design

We conducted a sequential explanatory mixed-methods cross-sectional study, using quantitative data from a facility assessment to enrich our qualitative inquiry. The qualitative study adopted an ontological approach which focuses on understanding the nature of participants’ realities, experiences, and perceptions. This was a formative study for a quality improvement initiative implemented by The AIDS Support Organisation (TASO).

### Study setting

**Fig. 1:**
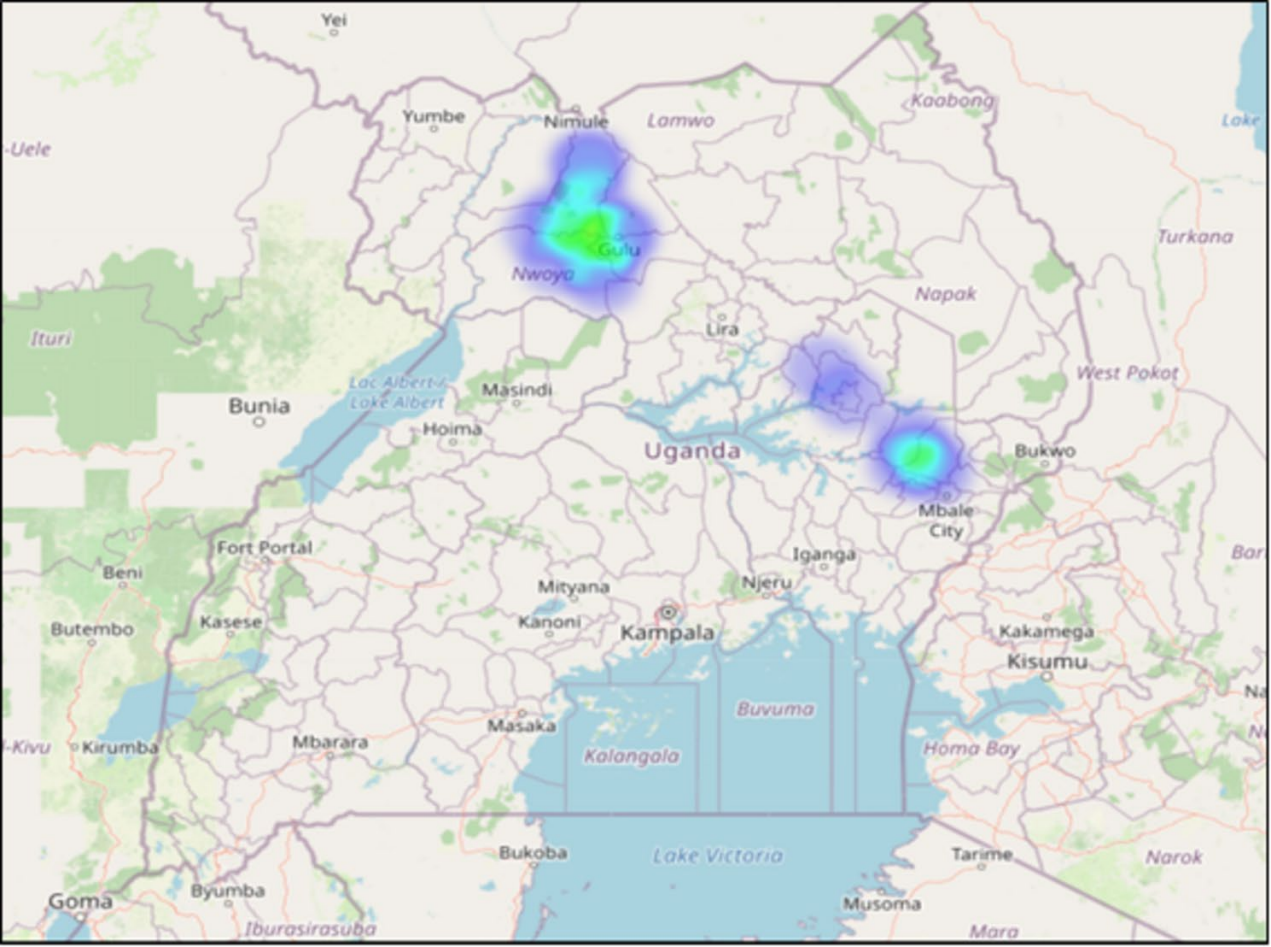
Map of Uganda showing data collection locations

The study was conducted among six city and district local governments, twenty health facilities and their catchment communities in two regions of Uganda (Acholi and Teso). The regions were selected purposively because of their higher-than-national average prevalence of hepatitis B infection based on a previous national-level survey [14]. Figure 1 below is a heat map of the locations where data collection took place.

#### Acholi region

We surveyed three out of nine political units in the region: Gulu City, Nwoya and Amuru districts. These included a total of ten health facilities: Gulu Regional Referral Hospital, TASO Gulu Special Clinic (Gulu City), Anaka General Hospital (GH), Koch Goma HCIII, Koch Lii HCIII (Nwoya district), Atiak HCIV, Pabbo HCIII, Oberabic HC III and Otwee HCIII (Amuru District) [15].

#### Teso region

The political units surveyed were four: Soroti City, Soroti District, Kumi and Bukedea Districts. We included ten facilities: Soroti Regional Referral Hospital, Princess Diana HCIV and TASO Soroti Special Clinic (Soroti city), Tiriri HCIV (Soroti district), Atutur GH, Kumi HCIV, Mukongoro HCIII (Kumi District), Kidongole HCIII, Bukedea HCIV and Kachumbala HCIII (Bukedea district) [15].

### Study population

For the quantitative component, observations were conducted at all the twenty health facilities, while for the qualitative component, the study population included key informants from the Ministry of Heath STIs/AIDS Control division – eMTCT program, district and city health team members involved in the delivery of HIV, immunisation, maternal and child health services, and health facility unit heads. Participants for focus group discussions were pregnant and lactating mothers attending antenatal or young child clinics at the study health facilities, sexual partners of pregnant and lactating women, and community health workers (CHWs) involved in MCH activities. All individuals available at the time and venue of data collection and provided written informed consent were eligible to participate in the study.

### Sample size and sampling method

All study participants were purposively selected. For focus group discussions, we invited participants through a group sensitization talk at antenatal clinic waiting areas, We enrolled eligible and willing participants consecutively until we had raised eight participants to constitute a group. Key informants were identified through a snowball approach and were recruited until information saturation.

### Data collection

Data collection took place in July – August 2024. For the quantitative component, we visited 20 health facilities and used a 28-item checklist to assess the availability of trained staff, health registers, laboratory commodities and drugs, service availability, utilisation, and leadership and governance. We used a checklist to document evidence of availability of services and commodities for triple elimination (Supplementary file 1). The checklist was adapted from the WHO service availability and readiness assessment tool (Tracer indicators for communicable disease service availability and readiness) [13].

For the qualitative component, we conducted key informant interviews and focus group discussions using semi-structured interview guides, with open ended questions. The interview tools were specifically developed for the study (Supplementary file 2). The [16] interviews were conducted in English or the popular local language (Luo in Acholi region and Ateso in Teso region), by trained research assistants. The research assistants and respondents met at pre-agreed times and locations that assured privacy. We recorded the interviews and transcribed them verbatim at the end of the day. We also conducted focus group discussions among pregnant and lactating women attending antenatal clinics at the study facilities, partners of pregnant and lactating women and community health workers. Trained research assistants moderated the focus group discussions in both English and the predominant local languages by region, using translated focus group discussion guides. We recorded the focus group discussions using audio recorders and transcribed the proceedings verbatim.

### Data analysis

For the quantitative data, we determined the proportion of items available per facility and converted this into a percentage, then computed a mean percentage score for all sites, using Microsoft Excel. We used findings from the quantitative data to enrich the interviews and focus group discussions. We conducted the qualitative analysis manually. Two coinvestigators read the transcripts, generated codes based on the WHO health systems strengthening framework, searched the data for themes and performed deductive coding. We extracted quotes that best illustrated the themes.

## Results

### Assessment of service availability

In Table 1, below, we present percentage service and commodity availability for each of the sites. Site scores per parameter assessed are summarized in supplementary file (Supplementary file 3 – Service and commodity availability assessment).

**Table 1:** Percentage service and commodity availability by health facility

Facility (location)	Total score/28	%
Acholi Region		
Facility A (semi-urban)	18	64.3%
Facility B (semi-urban)	21	75.0%
Facility C (urban)	22	78.6%
Facility D (rural)	15	53.6%
Facility E (rural)	14	50.0%
Facility F (rural)	20	71.4%
Facility G (rural)	13	46.4%
Facility H (rural)	17	60.7%
Facility I (semi-urban)	17	60.7%
Facility J (urban)	16	57.1%
Average	17.3	61.8%
Teso Region		
Facility K (semi-urban)	18	64.3%
Facility L (rural)	17	60.7%
Facility M (semi-urban)	19	67.9%
Facility N (rural)	17	60.7%
Facility P (urban)	20	71.4%
Facility Q (rural)	16	57.1%
Facility R (urban)	20	71.4%
Facility S (urban)	22	78.6%
Facility T (urban)	15	53.6%
Facility U (semi-urban)	21	75.0%
Average	18.5	66.1%

The sites with the highest readiness scores were regional referral hospitals for both regions, scoring similar readiness percentages (78.6%). At the time of data collection, all facilities, save for one, had had staff trained in triple elimination. While all facilities were accredited to provide anti-retroviral therapy for HIV treatment and prevention of mother-to-child transmission of HIV, they all had no stock of oral tenofovir disoproxil fumarate (TDF, 300 mg) tablets for Hepatitis B monotherapy, kits for DNA PCR testing of HIV-exposed infants (apart from one) and co-trimoxazole syrup for HIV prophylaxis among HIV exposed infants.

## Key informant interviews and focus group discussions

### Participant characteristics

#### Key informants

The study included a total of 10 key informants, seven of whom were aged 39–48 years, and the others were aged 29–38 years. There were no participants under 29 or over 49 years. Of the informants, six were females and four were males, two were from the ministry of health (national level), five were district health team members while three were health unit heads; providing a diverse but focused sample for this qualitative study.

#### Focus group discussion participant characteristics

The focus group discussions included 43 participants. 21, were aged 18–28 years, followed by 11 participants aged 28–38 years and 10 participants aged 38–48 years. Only one participant was aged 48 years or older. Focus group discussions consisted of 30 women and 13 men overall. We conducted a total of six focus group discussions (three in each of the two regions), two consisted of partners of pregnant and breastfeeding women, and two consisted of community health workers.

### Facilitators of the provision of triple elimination services

We present the facilitators of the provision of triple elimination services, by domain of the WHO health systems strengthening framework.

#### Leadership and governance

In the leadership and governance domain, respondents noted that the Ministry of Health had provided standard operating procedures and guidelines for the implementation of triple elimination services, which demonstrated strong national leadership for the triple-elimination initiative. The respondents recounted a ministry-of-health-led district and facility training on the revised consolidated national guidelines for HIV testing, prevention and treatment, that clearly provided for integration of triple screening and treatment for HIV, syphilis and hepatitis B within the maternal and child health clinics. At the district level, respondents noted that regular supportive supervision of facilities by district health teams was an enabler for the implementation of the triple elimination program. As one member noted,

In most cases, we do integrate supportive supervision whereby a team of City Health Office members go per facility as scheduled which is always conducted quarterly.

Fora for mutual accountability for program results were found also to facilitate the implementation of the triple elimination services. One district health team member noted,

*“We have a forum where we coordinate all health facility teams and provide guidance on program implementation”.*

We also learnt that the Ministry of Health collaborated with the Uganda AIDS Commission to develop and disseminate a handbook to be used by community leaders to disseminate information on PMTCT, “a PMTCT Handbook for Community Leaders.”

#### Healthcare financing

Off-budget financing for the implementation of triple elimination services was reported by district and facility-level stakeholders as critical to closing gaps in service delivery. She noted,

*Our implementing partner directly funds outreaches and stipends for mentor mothers to follow up mother-baby pairs under the eMTCT program*.

#### Health workforce

An important facilitator of the implementation of the triple elimination services is that nearly all facilities had their facility health workers trained on the triple elimination package during the recent roll-out of the revised consolidated guidelines for testing, prevention and treatment of HIV in Uganda.

*“We do refresher training of the midwives and the nurses such that at least they have up-to-date knowledge about these interventions” – District eMTCT focal person.*

*“MCH focal persons are knowledgeable and skilled enough to handle these particular activities” – DHO.*

In addition, respondents reported that the delivery of triple elimination services was boosted by the availability of motivated community health workers: mentor mothers and village health team members. These educate communities on diverse health topics and deliver community health services such as HIV testing, follow-up of mother-baby pairs for ART prophylaxis and early infant HIV diagnostic testing (EID).

*“We have a peer mother and a mentor mother who helps directly in those EID departments. She is in charge of tracking mother’s appointments*,* their expected dates*,* viral first PCR*,* and second PCR.…. giving these groups antenatal sessions and then even following them up when they are lost*,* tracking them from the community.” –* Health facility in-charge.

#### Medicines, commodities and vaccines

Our assessment also found the availability of stock-monitoring tools such as the real-time ARV stock monitoring system (RASS), was an enabler for district medicines supervisors and HIV focal persons to identify over- and under-stocked facilities to inform re-distribution efforts.

Furthermore, community members reported that the introduction of HIV self-test kits has contributed to improved access to HIV testing. Through distributing these kits, community health workers were able to increase access to HIV testing services among pregnant and lactating mothers who experienced structural barriers to accessing these services such as overlapping stigma, and unfavourable health facility opening times for HIV testing services.

#### Health information systems

The district health team members commended the regional PEPFAR-funded implementing partners for supporting data collection and utilisation for program improvement. They noted that the partners ensured timely distribution of paper-based reporting tools to facilities, conducted mentorship on the use of the tools, and facilitated district-led data quality audits. Owing to this support, the capacity of district biostatisticians has been built, to be able to summarise data from various health facilities and generate colour-coded dashboards to facilitate decision-making for service improvement.

#### Health services

The respondents noted that the implementation of community outreaches to reach pregnant women, lactating mothers and their families with services has been a critical facilitator. In the Acholi region, community Hepatitis B vaccination campaigns have been pivotal in increasing service coverage and uptake, as one DHT member noted, *“With Hepatitis B*,* in previous years we have had campaigns.*”

Furthermore, to ensure that demand for services is generated, health facility teams work with community mobilisers to provide information and address any myths and misconceptions. Some of the respondents noted that the engagement of religious and cultural leaders has been beneficial in addressing community hesitancy to utilize health services. One district health team member mentioned that,

*“We are (working with) the VHTs*,* the local counselor*,* and the “Rwot kweeri” - normally the “lower leadership” and we normally collaborate with them*,* they help us to mobilize*,* they help us to give all this information when are going there…*,*” –* DHT member.

Finally, the integration of syphilis screening and sensitization within antenatal clinics has contributed to increased coverage of syphilis services.

### Barriers to the provision of triple elimination services

#### Leadership and governance

Inadequate engagement of district health teams in triple elimination services was highlighted as an important barrier. Respondents noted that strong engagement of district health teams in these services was essential for quality service delivery, given the country’s decentralised nature of health system governance. One respondent argued that differences in district performance across several health indicators are largely attributed to the level of involvement of district health teams. She further noted, that,

…*there’s a tendency to leave some of those programs totally to be run by the implementing partners: we’ve seen some changes in some of the districts where we’ve been able to engage the DHO and ADHO and once they take ownership*,* we see better results and improvement.*

#### Healthcare financing

Respondents noted inadequate financing for triple elimination services. The district leaders noted that funding for the integration of Hepatitis B and syphilis-specific services in the established HIV and maternal child health platforms is sub-optimal. One district leader noted that often, funds are restricted to disease-specific programs, constraining their use for other disease areas. One district health team member noted, *“I don’t have a budget to move for supervision”*.

#### Health workforce

Regarding the health workforce, the respondents noted that the number of facility health workers is low compared to the work demands of delivering integrated care. One district health team member noted,

*I would give a case of HCIIs*,* they have a limited number of staff to do all the integration in terms of offering all the services.*

*“… inadequate staffing…*,* staff rotations; organized absenteeism (and) that really increases the amount of work they have to do. So*,* if you’re not very well motivated*,* you end up filling up some tools and leaving the others. And that again impacts on the quality of our reports*,* because we have documentation gaps.” –* MoH respondent.

*“It (service integration) would work but we usually have challenges of staff.” - CHW.*

Furthermore, respondents reported that whereas community volunteers have bridged the health workforce numerical gap, the lack of adequate supervision could compromise the quality of care provided. One district health team member noted,

*a lot of work is left to community linkage facilitators…the nurses and midwives concentrate on other elements*,* leaving the bulk of work to the volunteers*,* you know volunteers are also overwhelmed so sometimes those critical services that they also need to offer*,* you find that those services are not offered*,* and you find that the data won’t be also captured.*

One of the DHT members also noted that,

*We don’t know how to make EPI/EID integration work: I don’t know how we should plan for it unless we sit down… and plan.*

In this response, the DHT member suggests that despite the fact that the ministry of health has provided guidance on a package of services for EPI/EID integration (in the national guidelines), other contextual factors such as human resource constraints may limit the components of the package that can be delivered. As such, in some contexts, there is need for additional support with staff training and care re-organisation to assure holistic care for the infants.

Apart from health personnel shortages, respondents also noted that negative attitudes and inadequate skill among some health workers may also hinder the delivery of triple elimination services, noting that, “*some people are not result-oriented and don’t like their work*” and “*some health workers have knowledge gaps in management of Hepatitis B and syphili*s.” These sentiments corroborate findings on staffing gaps, transfers, relocation and exits of trained staff.

#### Medicines, commodities and vaccines

Nearly all respondents reported challenges in the availability of medicines, commodities and vaccines for triple elimination. One health facility head reported that his facility had not had syphilis test kits for more than a month. The following quotes reflect the voices of the respondents regarding the availability of supplies for testing, treating and prevention of HIV, syphilis and Hepatitis B.

*“Even these hepatitis B test strips*,* most times we don’t have. We don’t have the test kits but we know we are supposed to do it (testing for Hepatitis B)” –* DHT member.

*“For example*,* clients come and the regimens for Hepatitis B are not available. So*,* we have been referring either to Gulu Regional Referral Hospital (GRRH) or to the fourth division (Bardege HCIII) and sometimes at Lacor”.* – Health facility head.

One of the respondents noted that sometimes, the commodity and medicines stock-outs at health facility levels are due to challenges in fore-casting and requisitioning stocks from the central medicine warehouses. This could be worse for health center II’s that only started ordering for ART commodities for provision of PMTCT services within the previous financial year. This was aimed at bringing PMTCT services closer to the communities, since these were previously provided at higher level facilities.

#### Health information systems

Participants noted several barriers to quality reporting and data use for decision-making. Key among these were system downtime, heavy reporting requirements, incomplete data capture at service delivery points and inadequate reporting tools. A city health team member recounted,

*“The city has also been struggling with the data in the system. We have challenges of accessing dhis2.” –* DHT member.

*“So*,* this is evident that many times we intervene but then due to laxity*,* we don’t record what we do in the long run*,* what is captured may not be a true reflection of what we do*,* and that in a way impacts on how we plan on the intervention.” –* Health facility head.

On reporting requirements, one DHT member noted that “…. *we have the (HMIS) 106*,* a report that is a mammoth of data elements. Also*,* on a monthly basis*,* we have a section that talks about Hep B which has over ten indicators to report on. but based on the report*,* you find that out of 34 facilities*,* less than 5 can report on the Hep B testing.”*

#### Health services

Of health services, participants noted missed opportunities for delivery of triple elimination services because the models used do not respond to the challenges faced by a majority of the individuals targeted for the services, and the demand generation interventions are inadequate to address myths and misconceptions. Whereas the ministry of health is implementing a strategy to capacitate health center II’s to provide PMTCT services, one respondent reported that community members may not be aware of this shift, and thus, may not fully utilize the services. In addition, one of the pregnant participants in a FGD reported that,

*if I don’t see a protruding tummy, I can’t trust that the pregnancy is going to stay long; (what if I start going for ANC and) it doesn’t turn out as we expected?*

*“Mothers often complain about receiving drugs for only one month and face long distances to reach the facility. Additionally*,* there is a lack of transport available to the facility. I hope that EMTCT services can be integrated into more comprehensive packages to address these issues.” –* DHT member.

## Discussion

This study identified several facilitatorss and barriers to services for the triple elimination of vertical transmission of HIV, Hepatitis B and syphilis. Whereas the Ministry of Health has revised guidelines and established a national plan for triple elimination, stewardship at district and facility levels remains problematic in some districts. There is a suboptimal number and limited competence of human resources available to deliver the triple elimination package despite reports of several training activities having been delivered. Equally, frequent and long stock-outs of Hepatitis B birth dose vaccines, testing commodities, and treatment disrupt care for pregnant women and their infants. Health service delivery models and demand creation activities are not optimal for addressing socio-cultural and other constraints to the uptake of services. Moreover, some of the health facilities assessed do not have most of the drugs and test kits required to deliver triple elimination services. There is a need to harness the facilitators and critically examine the barriers to the delivery of integrated triple-elimination services to accelerate progress towards global and national goals.

In this study, we confirmed that strong district leadership engagement and a coordination platform were important to ensure proper supervision of and mutual accountability for service integration at lower-level health facilities. This finding is supported by Karusa et al. who noted that a coordination mechanism that enables communication, alignment of plans and budgets and joint problem-solving was critical health system practice in fostering PMTCT integration in MCH services [15]. Just like Uwimana et al. from South Africa noted, acceleration of triple elimination services requires political will and strong leadership [17]. Therefore, interventions aimed at improving integration of triple elimination of vertical transmission of HIV, syphilis and Hepatitis B should empower district health offices to champion local coordination platforms. These will ensure that national ministry efforts to develop and disseminate evidence-based policies and guidelines are translated into tangible health outcomes.

Berlacher et al. highlighted the need for health worker training on new protocols as a key challenge to pMTCT service integration in MCH clinics [16]. In our study, we found that whereas training had been done for health workers on the triple elimination guidelines, the numbers of those trained were inadequate to provide the scope of services within the triple elimination package. As such, districts and health facility teams relied on community health workers to mitigate the gap in health worker numbers. However, this shifting of tasks was encumbered by inadequate supervision of the community health workers. Our findings are similar to those of Smith et al., who examined the role and experiences of community health workers in Malawi [18]. Their study highlighted that task shifting to community health workers was often hindered by inadequate supervision, which impacted the effectiveness of health service delivery. Furthermore, the same study showed that health workers in MCH clinics had a negative attitude to pMTCT, considering it a specialized area, beyond their mandate. While this was not the case in our study, our respondents also flagged negative health worker attitudes as a potential barrier to delivery of triple elimination services. There is therefore need to systematically address drivers of negative health worker attitudes to ensure successful service integration.

Our study found that inadequate stocks of drugs and testing commodities were an important barrier to the integration of triple elimination services. The respondents’ perspectives were supported by an availability assessment that showed suboptimal stock levels of antiviral drugs, and testing commodities. Similarly, the Kenyan study poised that one of the challenges to service integration was delays in laboratory results return, partly attributable to testing commodity stock-outs [16]. Availability of rapid test kits such as lateral flow assays facilitates point-of-care diagnosis and timely linkage to treatment and prevention services [19]. There is therefore need to ensure that such supplies are consistently available to minimize missed opportunities [20]. Effective stock monitoring tools could facilitate the identification of overstocked and understocked facilities, thereby informing redistribution efforts. This finding aligns with the WHO’s emphasis on the importance of effective procurement and supply management (PSM) systems in preventing stock-outs and overstocking of essential medicines. The WHO has thus recommended early-warning indicators to track the performance of national PSM systems and prevent frequent stock imbalances, that ought to be adopted [21].

We further found that the introduction of HIV self-test kits has improved access to HIV testing, particularly among pregnant and lactating mothers facing structural barriers such as overlapping stigma and inconvenient health facility hours. Community health workers distributing these kits have been instrumental in increasing testing uptake. Community health workers play a vital role in improving access to mothers and exposed infants through conducting peer-to-peer education, psychosocial support, community-based testing and follow up care [22]. The positive effect of CHW interventions is largely attributable to cultural and linguistic congruence with the service beneficiaries, influencing patient behavior and service acceptability [23]. CHW-led interventions for triple elimination are therefore critical among sub-populations that may face unique barriers such as adolescent mothers who face overlapping stigma, and are therefore less likely to know their HIV status at their first ANC visit, have lower use of ART, higher rates of loss-to-follow-up and higher mother-to-child transmission rates [24]. In our study, respondents concurred that implementing community outreaches, engaging community leaders, and integrating syphilis screening within antenatal clinics have been effective strategies to enhance service delivery among pregnant and lactating women. Therefore, to optimize triple elimination services coverage and utilization, it is worthwhile to capacitate CHWs, strengthen their supervision and ensure appropriate remuneration [25, 26].

PEPFAR-funded implementing partners supported data collection and utilization for program improvement. This support also resulted in district biostatisticians building capacity for data utilization to facilitate decision-making and improve service delivery. On the downside, we observed that increased requirements for documentation and reporting were a major challenge in achieving service integration. Similarly, increased requirements for documentation were also reported by Berlacher et al., as a key barrier to effective service integration [16]. Their study highlighted that increased documentation requirements posed challenges to service integration, as healthcare providers faced additional administrative burdens that impeded efficient service. These contrasting findings highlight the importance of balancing comprehensive documentation with user-friendly health information systems that enhance service integration without overburdening healthcare providers. However, since PEPFAR funding was pivotal in building robust systems for accurate reporting and quality service delivery, recent funding suspensions are likely to aggravate these gaps and make attainment of global triple elimination goals more elusive. There will be need to advocate for restoration of suspended funding and institution of alternative funding to consolidate the gains and sustain momentum.

### Strengths and limitations

To the best of our knowledge, this is the first study in our setting to document health system facilitators, barriers to and health facility availability of commodities for delivery of the triple elimination package. Whereas previous studies have documented challenges to integration of PMTCT for HIV in maternal and child health clinics, the findings of our study re-affirm that similar challenges persist and are negatively impacting integration of additional disease screening, treatment and prevention interventions for syphilis and hepatitis B services within the MCH services [3, 4, 10, 11, 15, 27, 28]. One limitation of our study is that it was conducted in regions with a high burden of hepatitis B, where the challenges of service integration are likely to be more prominent, and the findings unlikely to be generalized to lower burden settings. Nonetheless, health system improvements in these regions are likely to portend the most population benefit and return on investment.

## Conclusions

The global aspiration of service integration for triple elimination of HIV, syphilis and hepatitis B vertical transmission is facilitated by several health system factors such as trained health workers, district leadership engagement, the support of PEPFAR-partners and involvement of community health workers. However, significant health system barriers such as frequent drug and commodity stock-outs, low health worker numbers and limited district health team engagement, hinder optimal service delivery. Moreover, varying levels of integrated service readiness and availability exist among health facilities and between regions. There is need for implementation research to harness the opportunities and address context-specific persistent health system challenges, for service integration.

## Supplementary Information

Supplementary Material 1.

Supplementary Material 2.

Supplementary Material 3.

## Data Availability

Data is provided within the manuscript and supplementary information file 1.

## Abbreviations

AIDS: Acquired immune deficiency syndrome
ANC: Antenatal care (clinic)
ART: Anti-retroviral therapy
ARVs: Anti-retroviral drugs
CHAI: Clinton health access initiative
DBS: Dried blood spot
DHIS2: District health information systems (version 2)
DHT: District health team
DNA: Deoxyribonucleic acid
eMTCT: Elimination of mother-to-child transmission of HIV
EPI: Expanded program on immunisation
GH: General hospital
HBV: Hepatitis B Virus
HC: Health center
HepBsAg: Hepatitis B surface antigen
HIV: Human immunodeficiency virus
LC: Local council
MoH: Ministry of health
PCR: Polymerase chain reaction
PMTCT: Prevention of mother-to-child transmission of HIV
REC: Research ethics committee
RRH: Regional referral hospital
TASO: The AIDS support organisation
TDF: Tenofovir disoproxil fumarate
TWG: Technical working group
UNAIDS: United Nations Joint program on HIV/AIDS
UNCST: Uganda National council for science and technology
WHO: World health organization
YCC: Young child clinic
