# Supplementary material for: Facilitators, barriers and service availability for delivering integrated care for the triple elimination of HIV, syphilis and hepatitis B vertical transmission in Uganda: a multi-site explanatory mixed methods study
Source: BMC Health Serv Res. 2025 May 1;25:626. doi: 10.1186/s12913-025-12797-4 (PMC12044932; doi:10.1186/s12913-025-12797-4)
Supplement: Supplementary file 1 — Supplementary Material 1. [file 12913_2025_12797_MOESM1_ESM.docx]

Facility assessment checklist

|  | Facility | Response 1 = Yes, 0 = No |
| --- | --- | --- |
| 1 | Has the facility had adequate stock of HIV, Hepatitis B or syphilis drugs in the last 3 months? |  |
| 2 | Does the facility have stock of TDF monotherapy? |  |
| 3 | Does the facility currently have stock of NVP syrup? |  |
| 4 | Does the facility currently have stock of septrin syrup? |  |
| 5 | Does the facility currently have stock of Benzathine penicillin? |  |
| 6 | Does the facility currently have stock of Hepatitis B birth-dose vaccine? |  |
| 7 | Does the facility currently have stock of Hepatitis B surface antigen RDTs? |  |
| 8 | Does the facility currently have stock of DBS kits for HEI PCR testing? |  |
| 9 | Does the facility currently have stock of HIV-syphilis duo test kits? |  |
| 10 | Does the facility currently have stock of HIV Determine test kits? |  |
| 11 | Does the facility have staff trained in MoH triple elimination guidelines? |  |
| 12 | Does the facility provide counselling for Hepatitis B infected individuals? |  |
| 13 | Are HIV, syphilis and Hepatitis B screening services provided at ANC, L&D and PNC? |  |
| 14 | Does the facility treat mothers with Hepatitis B? |  |
| 15 | Does the facility offer NVP syrup, CPT prophylaxis and Hepatitis B vaccination to infants? |  |
| 16 | Does the facility integrate HIV EID in Hepatitis B EPI activities? |  |
| 17 | Does the facility provide antiviral agents for HIV and Hepatitis B treatment? |  |
| 18 | Does the facility provide syphilis treatment? |  |
| 19 | Is HIV-syphilis duo testing available to all pregnant and lactating mothers at this site? |  |
| 20 | Is Hepatitis B treatment eligibility testing, including liver function testing available at this facility? |  |
| 21 | Is abdominal ultrasonography available at this site? |  |
| 22 | Does the facility have job aides for Health workers on HIV, Hepatitis B and syphilis management? |  |
| 23 | Does the facility have IEC materials on HIV, syphilis and Hepatitis B? |  |
| 24 | Does the facility have data capture tools for reporting HIV, Hepatitis B and syphilis screening and treatment? |  |
| 25 | Does the facility team meet regularly to review PMTCT performance data? |  |
| 26 | Does the facility have staff trained in QI? |  |
| 27 | Does the facility have a HUMC to oversee operations and review performance? |  |
| 28 | Is the facility regularly supervised by the district health team? |  |
|  | Health facility total |  |
