# Supplementary material for: Facilitators, barriers and service availability for delivering integrated care for the triple elimination of HIV, syphilis and hepatitis B vertical transmission in Uganda: a multi-site explanatory mixed methods study
Source: BMC Health Serv Res. 2025 May 1;25:626. doi: 10.1186/s12913-025-12797-4 (PMC12044932; doi:10.1186/s12913-025-12797-4)
Supplement: Supplementary file 2 — Supplementary Material 2. [file 12913_2025_12797_MOESM2_ESM.docx]

**Appendix 4: Qualitative Guides**

Focus Group Discussion Guide (clients/service beneficiaries, sex partners, staff, CHWs/VHTs, HUMCs)

1. May you please tell us what you understand by Triple Elimination Package – or management of three conditions for eMTCT? *Probe for knowledge of the conditions, management and services*
2. What services do you receive/provide under the eMTCT in/at your district hospitals? At the health facility/or at the community?
3. What systems (opportunities) are in place at your facility/community to ensure provision of eMTCT services for chronic hepatitis B infection at this health facility/district?
4. Which tools, guidelines, or resources do you use in supporting eMTCT? Or during provision of eMTCT services at this facility, or at your community as CHWs?
5. What gaps/challenges/needs is this health facility/district/community facing in the provision of eMTCT services for HIV? *Probe for gaps along HSS building blocks.*
6. What challenges/gaps do you clients/service beneficiaries face in accessing eMTCT and TEP services?
7. What are some of the gaps that exist at individual, sex partner and community level that are barriers to access and uptake of eMTCT/triple elimination services?
8. What strategies, and recommendations would you like to make to any project that would like to support the area of eMTCT, hepatitis B and syphilis at your health facility, community and personal level?

Key informant interview (KII) and in-depth interview (IDI) guides – MoH, DHO, In charges, HUMC

1. May you please tell us what you understand by Triple Elimination Package or management of three conditions for eMTCT?
2. What services do you provide through the eMTCT at your district hospitals, health facility or at the community? How are the eMTCT/triple elimination services being accessed at your district, facility and community?
3. What systems/opportunities, guidelines, tools and resources are in place to ensure provision of eMTCT services for HIV at national, district, health facility and community?
4. What challenges/barriers is MoH/district/health facility, community facing in the provision of eMTCT services for HIV?
5. What challenges/gaps is this health facility/district facing in provision of eMTCT services for teiple elimination? *Probe for gaps along HSS building blocks*
6. What are some of the gaps that exist at individual, sex partner and community level that are barriers to access and uptake of the eMTCT/triple elimination services?
7. Tell us about the quality improvement projects at the MoH, district, facility that are supporting the TEP indicators
8. What strategies, and recommendations would you like to make/suggest to a project that would like to support in the areas of triple elimination?
